# Dataset of growth cone-enriched lipidome and proteome of embryonic to early postnatal mouse brain

**DOI:** 10.1016/j.dib.2019.103865

**Published:** 2019-03-26

**Authors:** Anna Trzeciecka, Sanjoy K. Bhattacharya

**Affiliations:** Bascom Palmer Eye Institute, Department of Ophthalmology, University of Miami, Miami, FL 33136, USA

**Keywords:** Growth cone, Neuron, Forebrain, Developmental stages E18, P0, P3, P6, P9, Lipid profile, Protein profile, Mass spectrometry

## Abstract

A growth cone is a part of a neuron considered as a hub for axon growth, motility and guidance functions. Growth cones are thought to play a critical role during development of neurons. Growth cones also play a significant role in adult regeneration. Here, we present a dataset on the lipid and protein profiling of the growth cone-enriched fractions derived from C57BL/6J mice forebrains of developmental stage: E18, P0, P3, P6, and P9. For comparison, we analyzed non-growth cone membranes from the same samples. Lipid data is available at the Metabolomics Workbench [http://www.metabolomicsworkbench.org (Project ID: PR000746)]. Protein data is available at Proteomics Identifications (PRIDE) partner repository (PRIDE identifier PXD012134).

**Table:** Specifications table

Subject area	Biology
More specific subject area	Proteins, lipids
Type of data	Chromatograms, spectra, tables
How data was acquired	liquid chromatography Q-Exactive Orbitrap mass spectrometry
Data format	Raw, analyzed, filtered
Experimental factors	Brain subcellular fraction, age
Experimental features	Growth cones were enriched by sucrose gradient ultracentrifugation from embryonic and early postnatal C57BL/6J mice forebrains. Non-growth cone membranes from the same samples were collected for comparison. Lipids and peptides were analyzed by untargeted LC-MS/MS.
Data source location	Bascom Palmer Eye Institute, Miller School of Medicine at University of Miami, Miami, FL 33136, USA
Data accessibility	The Metabolomics Workbench – Project ID: PR000746, https://doi.org/10.21228/M87X0C PRIDE – accession number: PXD012134, doi: Not applicable [PRIDE accession review details-Username: reviewer33503@ebi.ac.uk Password: QxZ1t6YZ, Website: http://www.ebi.ac.uk/pride]
Related research article	[1].

**Table:** 

**Value of the data** • The dataset complements existing resources on the growth cone composition. Lipid and protein time-course profiling analysis of growth cone-enriched and non-growth cone membrane fractions may be of interest to developmental and regenerative neuroscience research. • The dataset can be used to derive information on the growth cone composition throughout the tested period or at the specific developmental time-point. The data can be integrated with other omics approaches. Hypotheses on the specific lipid and/or protein changes and their potential role in growth cone biology may be generated from this dataset and form the basis of functional studies. • The data can be used to create peptide/lipid spectral libraries for targeted proteomic/lipidomic experiments.

## 1 Data

**Fig. 1:**
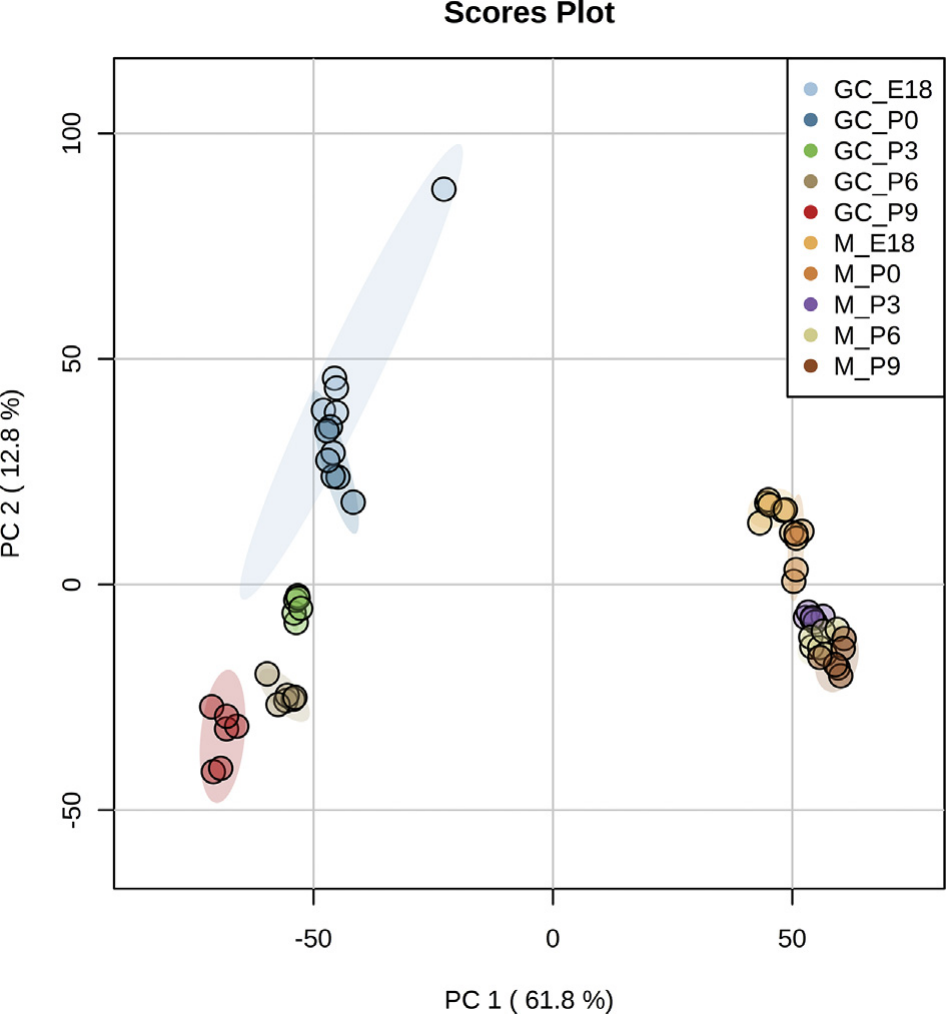
Principal component analysis (PCA) of lipid profiling data. Data from Supplementary Table S1 has been filtered to lipid species detected in at least 4 biological replicates. Missing data was replaced by column min, followed by quantile normalization and log transformation (glog). Samples are plotted in 2 dimensions using their projection onto the first 2 principal components (in brackets % of total variance explained).

**Fig. 2:**
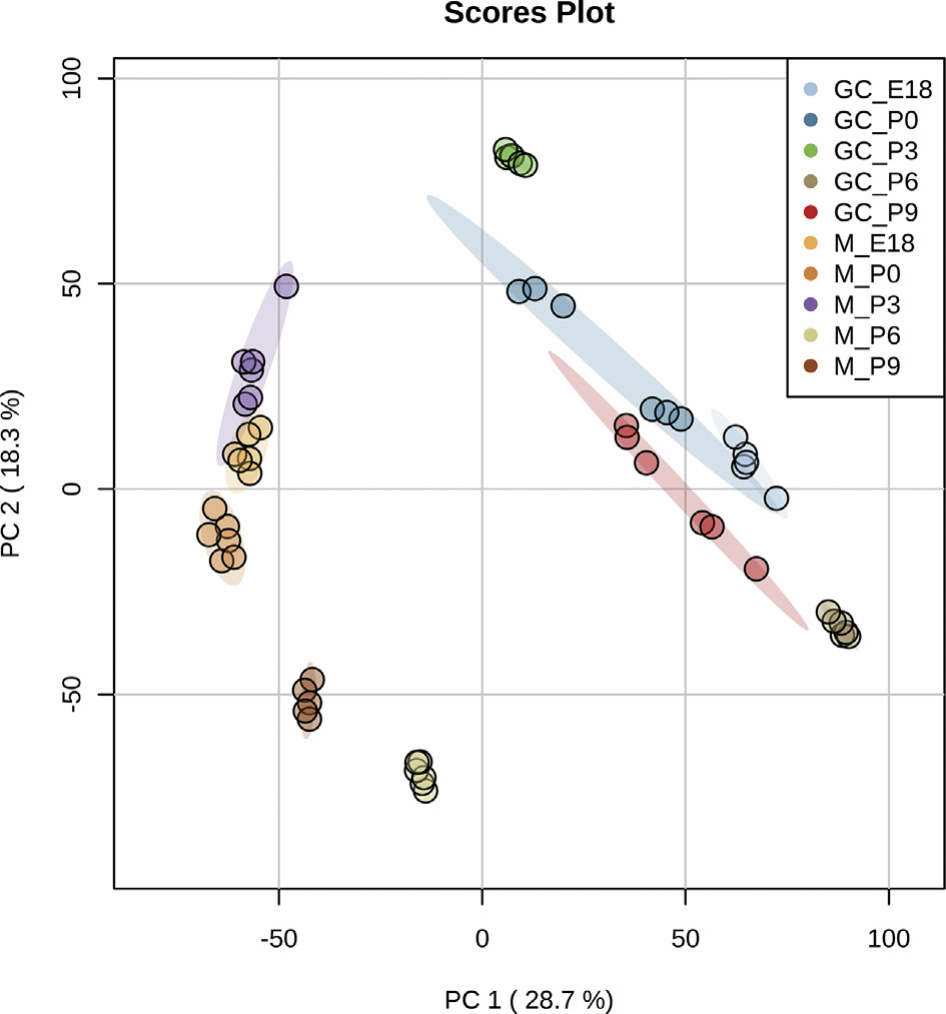
Principal component analysis (PCA) of protein profiling data. Data from Supplementary Table S2 has been filtered to protein accessions detected in at least 4 biological replicates. Missing data was replaced by column min, followed by quantile normalization and log transformation (glog). Samples are plotted in 2 dimensions using their projection onto the first 2 principal components (in brackets % of total variance explained).

Here, we carried out lipid and protein profiling of the growth cone (GC)-enriched fractions derived from forebrains of E18 – P9 C57BL6/J mice. Lipids were extracted using chloroform, methanol and water mixture to obtain phase separation. We then used untargeted liquid chromatography Q-Exactive Orbitrap tandem mass spectrometry (LC-MS/MS) for lipid profiling. Peak extraction, identification, relative quantification, and alignment were performed using Lipid Search 4.1 software. The list of identified lipids is provided in Supplementary Table S1. In parallel, protein samples were reduced, alkylated and digested using trypsin and Lys-C proteases, followed by LC-MS/MS. Proteome Discoverer 2.2 was used for bioinformatics analysis. The list of identified proteins is provided in Supplementary Table S2. Samples clustering is presented in Fig. 1 and Fig. 2 for lipid and protein profiling data, respectively.

## 2 Experimental design, materials, and methods

### 2.1 Brain tissue collection and subcellular fractionation

All animal procedures were performed in accordance with the ARVO Statement for the Use of Animals in Ophthalmic and Vision Research and policies of the UM Institutional Animal Care & Use Committee (IACUC). Forebrains of E18, P0, P3, P6, and P9 C57BL6/J mice were fractionated by sucrose gradient ultracentrifugation into growth cone-enriched (GC) and non-growth cone membrane (M) fractions as described previously [1], [2], [3], [4], [5]. Protein concentrations were determined using BCA assay (Thermo Fisher Scientific, Waltham, MA), according to manufacturer's instruction. For samples list, see Table 1.

**Table 1:** Samples list.

Fraction	Age	Biological replicate
growth cone-enriched membranes (GC)	E18	1–6
	P0	1–6
	P3	1–6
	P6	1–6
	P9	1–6
non-growth cone membranes (M)	E18	1–6
	P0	1–6
	P3	1–6
	P6	1–6
	P9	1–6

### 2.2 Lipid profiling

#### 2.2.1 Sample preparation

6 mL of methanol (LC-MS grade) and 3 mL of chloroform (LC-MS grade) were added to each sample (100 μg of total protein). After 2 min vigorous vortexing and 2 min sonication in an ultrasonic bath, the samples were incubated at 48 °C overnight (in borosilicate glass vials, PTFE-lined caps). The following day, 3 mL of water (LC-MS grade) and 1.5 mL of chloroform were added, samples vigorously vortexed for 2 min and centrifuged at 3000 RCF, 4 °C for 15 min to obtain phase separation. Lower phases were collected. Remaining samples (upper and interphases) were re-extracted by addition of 4.5 mL of chloroform and centrifugation at 3000 RCF, 4 °C for 15 min to obtain phase separation. Lower phases from both extractions were combined and dried in a centrifugal vacuum concentrator. Samples were stored at −20 °C until reconstituted in 150 μL of chloroform:methanol (1:1) before mass spectrometric analysis.

##### 2.2.1.1 High-performance liquid chromatography (HPLC)

Reversed-phase chromatographic separation utilized Accela Autosampler (Thermo), Accela 600 pump (Thermo) and Acclaim C30 column: 3 μm, 2.1 × 150 mm (Thermo). The column temperature was maintained at 30 °C and tray temperature at 20 °C. Solvent A was composed of 10 mM ammonium acetate (LC-MS grade) in 60:40 methanol:water (LC-MS grade) with 0.2% formic acid (FA; LC-MS grade). Solvent B was composed of 10 mM ammonium acetate with 60:40 methanol:chloroform with 0.2% FA. The flow rate was 260 μL/min, and the injection volume was 10 μL. The gradient was 35–100% solvent B over 13.0  min, 100% solvent B over 13.0–13.8 min, 100–35% solvent B over 13.8–14.5 min, 35% solvent B over 14.5–18.0 min, 0% solvent B over 18.0–20.0 min.

#### 2.2.2 Mass spectrometry

The Q Exactive (Thermo) mass spectrometer was operated under heated electrospray ionization (HESI) in positive and negative mode separately. The spray voltage was 4.4 kV, the heated capillary was held at 310 °C (negative mode) or 350 °C (positive mode) and heater at 275 °C (positive mode). The S-lens radio frequency (RF) level was 70. The sheath gas flow rate was 30 (negative mode) or 45 units (positive mode), and auxiliary gas was 14 (negative mode) or 15 units (positive mode). Full scan used resolution 70,000 with automatic gain control (AGC) target of 1 × 10^6^ ions and maximum ion injection time (IT) of 100 ms. Data-dependent MS/MS (top10) were acquired using the following parameters: resolution 17,500; AGC 1 × 10^5^; maximum IT 75 ms; 1.3 m/z isolation window; underfill ratio 0.1%; intensity threshold 1 × 10^3^; dynamic exclusion time 3 s. Normalized collision energy (NCE) settings were: 15, 30, 45, 60, 75, 90 (in positive and negative mode separately; total 12 runs per sample).

##### 2.2.2.1 Lipid identification and quantification

Lipid identification and relative quantification were performed using LipidSearch 4.1 software (Thermo). The search criteria were as follows: product search; parent m/z tolerance 5 ppm; product m/z tolerance 5 ppm; quantification: m/z tolerance 5 ppm, retention time tolerance 1 min. The following adducts were allowed in positive mode: +H, +NH4, +H—H2O, +H—2H2O, +2H, +Na, +K and negative mode: H, +HCOO, +CH3COO, -2H, —Cl. All classes were selected for search. LipidSearch nomenclature is used (Table 2).

**Table 2:** LipidSearch nomenclature of the identified lipid species.

Group	ClassKey	Lipid name
phospholipid	BisMePA	bismethyl phosphatidic acid
	dMePE	dimethylphosphatidylethanolamine
	LdMePE	lysodimethylphosphatidylethanolamine
	LPC	lysophosphatidylcholine
	LPE	lysophosphatidylethanolamine
	LPEt	lysophosphatidylethanol
	LPG	lysophosphatidylglycerol
	LPI	lysophosphatidylinositol
	LPS	lysophosphatidylserine
	PA	phosphatidic acid
	PAF	platelet-activating factor
	PC	phosphatidylcholine
	PE	phosphatidylethanolamine
	PEt	phosphatidylethanol
	PG	phosphatidylglycerol
	PI	phosphatidylinositol
	PIP	phosphatidylinositol
	PMe	phosphatidylmethanol
	PS	phosphatidylserine
sphingolipid	Cer	ceramide
	CerP	ceramide phosphate
	SM	sphingomyelin
	So	sphingosine
glycosphingolipid	CerG1	hexosyl ceramide
	CerG2	dihexosyl ceramide
	GD1a	ganglioside
	ST	sulfatide
cardiolipin	CL	cardiolipin
neutral glycerolipid	MG	monoglyceride
	DG	diglyceride
	TG	triglyceride
steroid	ChE	cholesterol ester
	ZyE	zymosterol
coenzyme	Co	coenzyme
fatty esters	AcCa	acyl carnitine
glycoglycerolipid	MGMG	monogalactosylmonoacylglycerol
	MGDG	monogalactosyldiacylglycerol
	DGMG	digalactosylmonoacylglycerol
	SQMG	sulfoquinovosylmonoacylglycerol
	SQDG	sulfoquinovosyldiacylglycerol

#### 2.2.3 Data processing

Positive and negative mode identifications at different NCE were aligned in LipidSearch, allowing calculation of unassigned peaks. The following settings were applied: product search; alignment method max; retention time tolerance 0.1 min; filters: top rank, main isomer peak; M-score 5; molecular lipid identification grade: A-B (A: lipid class and fatty acid completely identified or B: lipid class and some fatty acid identified).

### 2.3 Protein profiling

#### 2.3.1 Sample preparation

100 μg of proteins were precipitated with 4 vol of ice-cold acetone overnight at −20 °C. The following day, proteins were pelleted by centrifugation (15  min, 4 °C, 18,000 RCF). Pellets were resuspended in 20 μL of 0.2% ProteaseMAX (Promega)/50 mM ammonium bicarbonate (ABC). 50 mM ABC was added to a final volume of 93.5 μL. Proteins were reduced with dithiothreitol (DTT): 1 μL of 0.5 M (final concentration ∼5 mM) for 20 min at 56 °C. Next, proteins were alkylated using iodoacetamide (IAA): 2.7 μL of 0.55 M (final concentration ∼15 mM) for 15 min at room temperature in the dark. 1 μL of 1% ProteaseMAX/50 mM ABC with 4 μg of Trypsin/Lys-C mix (Promega; 1:25 trypsin-to-protein ratio) was added. Digestion was carried out in a total volume of 100 μL (1 μg of total protein/μL) for 4 h at 37 °C. Samples were adjusted to 300 μL of 0.1% trifluoroacetic acid/50 mM ABC. Pierce High pH Reversed-Phase Fractionation Kit (Thermo) was used to separate peptides into eight fractions (according to manufacturer's instruction). We also collected flow-through, wash (water) and an additional 100% acetonitrile (ACN) elution fraction (total: 11 fractions). Fractions were dried in a centrifugal vacuum concentrator at 45 °C and then stored at −20 °C. Before LC-MS analysis, peptides were re-suspended in 30 μL of 0.1% FA in water.

#### 2.3.2 Ultra-high performance chromatography (UHLPC)

The chromatographic separation utilized EASY-nLC 1000 system (Thermo) and Acclaim PepMap RSLC 75 μm × 15 cm, nanoViper column (Thermo). Solvent A was composed of 0.1% FA in water (LC-MS grade). Solvent B was composed of 0.1% FA in ACN (LC-MS grade). The flow rate was 260 μL/min and injection volume was 10 μL. The gradient was 2–5% solvent B over 5.0  min, 5–60% solvent B over 5–70  min, 60–98% solvent B over 70–98  min, 98% solvent B over 98–118 min, 98-2% solvent B over 118–120 min.

#### 2.3.3 Mass spectrometry

The Q Exactive (Thermo) mass spectrometer was operated under heated electrospray ionization (HESI) in positive mode. The spray voltage was 1.8 kV, the heated capillary was held at 250 °C, and the S-lens radio frequency (RF) level was 70. Full scan (m/z 150–2000) used resolution 70,000 with automatic gain control (AGC) target of 1 × 10^6^ ions and maximum ion injection time (IT) of 100 ms. Data-dependent MS/MS (top15) were acquired using the following parameters: resolution 17,500; AGC 1 × 10^5^; maximum IT 200 ms; 1.3 m/z isolation window; NCE 28. Underfill ratio was set to 0.1%; intensity threshold to 5 × 10^2^; dynamic exclusion time to 20 s; peptide match to preferred; charge exclusion to unassigned and 1.

#### 2.3.4 Protein identification and quantification

The acquired raw files were analyzed with Proteome Discoverer 2.2 (Thermo) using the SEQUEST HT engine. The data was searched against 83,916 Mus musculus entries (Swiss-Prot + TrEMBL, UniProt 8/14/2018). Search parameters included: precursor mass tolerance 10 ppm and 0.02 Da for fragments, 2 missed trypsin cleavages, oxidation (Met) and acetylation (protein N-term) as variable modifications, carbamidomethylation (Cys) as a static modification. Percolator PSM validation used the following parameters: strict FDR of 0.01, relaxed FDR of 0.1, maximum ΔCn of 0.05, validation based on q-value. For label-free quantification (LFQ), the Minora Feature Detector was used along with the Feature Mapper and Precursor Ions Quantifier. Data were filtered by peptide filter: medium (FDR 0.1) and high confidence (FDR 0.01) and protein filter: medium (FDR 0.1) and high confidence (FDR 0.01).

## Appendix A Supplementary data

Supplementary Table S1 List of identified lipids. Supplementary Table S1

Supplementary Table S2 List of identified proteins. Supplementary Table S2
